# Relationships between maternal emotional expressiveness and children's sensitivity to teacher criticism

**DOI:** 10.3389/fpsyg.2013.00807

**Published:** 2013-11-08

**Authors:** Ai Mizokawa

**Affiliations:** Faculty of Psychology, Meiji Gakuin UniversityTokyo, Japan

**Keywords:** Japanese, maternal expressiveness, negative emotion, sensitivity to teacher criticism, young children

## Abstract

Caregivers' emotional responses to children influence children's social and emotional development. This study investigated the association between maternal emotional expressiveness in the context of mother–child interactions and young children's sensitivity to teacher criticism. Sensitivity to teacher criticism was assessed among 53 Japanese preschoolers using hypothetical scenarios in which a puppet child representing the participant made a small error, and a puppet teacher pointed out the error. Self-report questionnaires were used to measure maternal expressiveness. The results demonstrated that negative maternal expressiveness toward one's own children was positively related to children's ratings of their own ability and negatively related to children's motivation to continue with the task after teacher criticism. Positive maternal expressiveness was not related to children's sensitivity to criticism. These findings suggest that children who have experienced more negative emotion from mothers may be more likely to hold negative beliefs about how others will respond to their behavior more generally. This may, in turn, lead to a defensively positive view of one's own abilities and a disinclination to persevere as protection from additional opportunities for teacher evaluation.

## Introduction

Starting in the early years of life, children are very sensitive to the emotional signals of others. For example, infants as young as 6 months of age follow the direction of adults' gaze (Scaife and Bruner, 1975; Butterworth and Jarrett, 1991). From about 12 months, infants often use their mothers' emotional cues to guide their own reactions in ambiguous situations (Saarni et al., 2006). A mother's emotional expressions serve not only as a guide to appropriate behavior in ambiguous situations but also as feedback to the child about the child's own behavior (Kelley et al., 2000). Hence, such maternal emotional reactions influence children's belief systems concerning the acceptability and reasonableness of their behavior (Halberstadt, 1991). The present study focused on how the emotional climate in families, especially the maternal emotional expressiveness in mother–child interactions, relate to children's responses to criticism (i.e., negative evaluation) in extra-familial situations.

Sensitivity to criticism is an important aspect of children's social and emotional development, and the style of maternal emotional expression may influence such sensitivity. Dweck and colleagues identified two distinct reactions to failure: helplessness patterns and mastery-oriented patterns (see Dweck, 1999). Children who respond to failure with helplessness tend to denigrate their intelligence and demonstrate drastically reduced expectations, negative emotions, less persistence, and deterioration in performance (Diener and Dweck, 1978, 1980). On the other hand, children who show a mastery-oriented response tend to remain focused on achieving mastery despite their present difficulties, maintain positive affect and self-assessment, and continue to exhibit constructive behavior (Diener and Dweck, 1978, 1980).

Issues related to children's vulnerability and motivation at school have been of major concern to parents, teachers, and researchers in recent years. Although individual differences in response to criticism were first identified in school-age children, later studies have demonstrated that some younger children show helpless responses after a salient failure (Heyman et al., 1992; Smiley and Dweck, 1994; Cutting and Dunn, 2002; Lecce et al., 2011, 2013). Previous studies have also reported that the different ways in which adults provide feedback have differential effects on young children's responses to criticism (Mueller and Dweck, 1998; Kamins and Dweck, 1999). Despite increasing interest in individual differences in sensitivity to evaluative feedback, very few studies have investigated how such sensitivity develops.

Of particular interest in the present study was the connection between maternal emotional expressiveness and children's sensitivity to criticism. The extant literature suggests that the emotional climate surrounding the mother–child relationship may affect the child's sensitivity to criticism (Ryan et al., 1994). The emotional climate in a family (i.e., maternal positive and negative emotional expressiveness) affects children's socio–emotional development. In general, the expression of positive emotions by parents is positively associated with children's social competence, whereas the expression of negative emotions tends to have the opposite effect on these developmental outcomes (e.g., Cassidy et al., 1992; Boyum and Parke, 1995; Halberstadt et al., 1999). Nonetheless, the findings for negative expressiveness are more complex than are those for positive expressiveness (Halberstadt et al., 1999). For example, the self-reported expression of negative emotions by mothers has been associated with children's less frequent use of prosocial display rules and greater reliance on self-protective ones (Jones et al., 1998). Moreover, the self-reported expression of negative emotions by mothers moderated associations between the personal distress and support seeking of children. Indeed, the association between distress and support-seeking was less negative when accompanied by higher levels of maternal negative-dominant emotional expressiveness (Goodvin et al., 2006). Longitudinal research has also found that the expression of negative emotions by mothers is negatively associated with children's constructive coping with daily stress (Valiente et al., 2004).

Berry et al. (2007) noted, “Negative beliefs about the self and others associated with insecure attachment would increase sensitivity to criticism and negative responses from others. Difficulties in regulating affect and subsequent hyperarousal associated with insecure attachment styles would also increase sensitivity to stress in the social environment” (p. 466). This suggestion implies that a positive climate surrounding a mother–child relationship would engender feelings of acceptance and security and form an internal model on which children base future relationships. It is also presumed that the child internalizes the style of mother–child emotional communication and uses this as a model for future relationships (cf. Bowlby, 1969/1982). Hence, maternal emotional expression in daily life presumably affects children's sensitivity to criticism in settings beyond the mother–child situation; that is, maternal emotional expression would be expected to influence how children feel and respond in situations in which they receive evaluative feedback from individuals other than their mothers. Moreover, uncontrolled parental expressions of negative emotion might model dysregulated behavior that children then imitate (Eisenberg et al., 2001).

Although the aforementioned previous studies did not directly involve maternal expressiveness and sensitivity to criticism, it is expected that children who have lived in a more negative climate would be more vulnerable to psychological damage than would those who have lived in a more positive climate because the former would experience the same criticism more negatively (Beardslee et al., 1998). The first hypothesis tested in the current study was that children with mothers who are highly positive in their expressiveness would be less sensitive to teacher criticism (i.e., demonstrate more positive emotional responses, rate their abilities more favorably, and express greater motivation to persevere after criticism). The second hypothesis was that children with highly negatively expressive mothers would be more sensitive to teacher criticism (i.e., demonstrate less positive emotional responses, rate their abilities less favorably, and express less motivation to persevere after criticism). Following Halberstadt et al. (1995), maternal self-reports were used to assess the emotional climate of children's living situations. To focus on maternal self-expressiveness in *mother–child contexts*, a modified version of the Self-Expressiveness in the Family Questionnaire (Halberstadt et al., 1995) was developed for the current study.

## Methods

### Participants

Fifty-three Japanese children (26 boys and 27 girls; mean age: 5.91 years; *SD* = 0.52 years) and their mothers (mean age: 38.25 years; *SD* = 4.15 years) participated in this study. Most mothers were full-time workers (81.1%), 15.1% were part-time workers, and 3.8% were temporarily unemployed. The mothers were generally highly educated: 1.9% had attended junior high school, 17.0% had attended high school, 15.1% had attended technical school, 9.4% had attended a 2-year college, 35.8% had attended university, 18.8% had attended graduate school, and 1.9% did not respond to this question. Most mothers were married (92.5%); 3.8% were single, and 3.8% were divorced. Children and mothers were recruited at three nursery schools in Tokyo, Japan. Although detailed data could not be obtained, most children spent about 8–10 hours per day at nursery schools and had been doing so since they were 3 to 12 months of age as most mothers worked full-time. Data from an additional 10 children were eliminated because their mothers did not complete the questionnaire.

### Procedure

#### Child measures

Each child was individually brought by the experimenter (the author) from the classroom to the experimental area in his/her school, where tasks measuring sensitivity to failure and criticism and vocabulary were administered.

***Sensitivity to failure and criticism*.** The experimenter read aloud three puppet-based stories. The children were instructed to pick a toy person from among four puppets to represent themselves and were introduced to another toy person who was to be their pretend teacher. The stories and procedures were developed based on Heyman's task (Heyman et al., 1992).

The children were presented with three analogous stories: story A, about painting; story B, about writing numbers; and story C, about trying to solve a jigsaw puzzle. The theme in stories A and B was the same: the main character (i.e., the participant) works hard on a task and then makes a small error. One of the stories (the failure story) ended at this point, and the other story (the criticism story) ended with the main character receiving criticism from a teacher. All children heard both versions of the story; the one without criticism was presented first. The failure story without criticism was used to establish a prejudgment baseline. To provide an opportunity for the toy teacher to offer praise, the children were instructed to have their toy person help another toy person with a puzzle; following this interaction, the toy teacher informed all the children that they did a great job and then offered a gracious apology for her earlier criticism (story C).

The following questions were posed after the failure (A or B) and criticism (B or A) stories: *(Q1) Emotional Response*: “I want to know how you feel about what happened with the [painting or number]. Do you feel happy or not? Do you feel sad or not? Do you feel angry or not?” One point was given for each positive response (happy, not sad, and not angry). The emotional response was indexed by summing the scores for all three emotion questions (scores ranged from 0 to 3). *(Q2) Ability Rating:* “Think again about everything that happened with the [painting or number]. Should you get a check (good) or a cross (not good) for what you did?” One point was given for a positive evaluation, and zero points were given for a negative evaluation (scores ranged from 0 to 1). Following the criticism story, two further questions were asked in addition to the first two questions: *(Q3) Motivation:* “Think again about everything that happened with the [painting or number]. If the teacher asked you to [paint a picture or write a number] again, would you do it or would you do something else nice instead?” One point was given when the children chose to do the activity again, and zero points were given when they choose to do something else (scores ranged from 0 to 1). *(Q4) Memory:* to ensure that any differences in responses to criticism were not merely the result of differences in the tendency to remember criticism, children were asked to describe what they remembered about the criticism story: “I'd like you to tell me all you remember about what the puppet teacher said to you in the story.”

***Vocabulary*.** The children's vocabulary was assessed using the Picture Vocabulary Test-Revised (PVT-R; Ueno et al., 2008), which required them to select the picture named by the experimenter from an array of four pictures.

#### Maternal measures

***Self-expressiveness in mother–child interactions*.** Maternal reports were obtained to determine the emotional climate of mother–child interactions. Mothers responded to 19 questions (addressing 10 positive and nine negative dimensions) included in a modified version of the Self-Expressiveness in the Family Questionnaire (SEFQ^1^; Halberstadt et al., 1995) in terms of the current mother–child situation. Each item was rated on a 4-point Likert scale (1 = not at all frequently, 4 = very frequently). The items in the modified version of the SEFQ are presented in the Appendix.

## Results

Preliminary analyses showed that maternal age, education, and marital status did not affect any of the analyses, and these variables were not considered further. Moreover, the data from the memory question in the sensitivity to failure and criticism task showed that all children passed this question, that is, they retained what the puppet teacher said in their memory when they answered the questions following the criticism story.

The 19-item questionnaire about maternal emotional expressiveness was submitted to principal-components factor analysis with promax rotation. A scree test revealed two factors, a 10-item positive factor and a nine-item negative factor, and this solution accounted for 41.07% of the variance. It should be noted that the internal consistency of the original version of the 40-item SEFQ was high for both positive (0.94) and negative (0.92) items. The 10 positive and nine negative items used in this study were derived from the original scale which contained 40 items.

For descriptive purposes, correlations among the study measures are presented at the top of Table 1. Means and standard deviations for the study measures are reported at the bottom of Table 1.To examine whether mother-reported emotional expressiveness differed by the sex of the child, a two-sample *t*-test was performed. No significant differences were found in mother-reported positive, *t*_(51)_ = 0.81, *ns*., or negative, *t*_(51)_ = −1.08, *ns*., emotional expressiveness according to the sex of the child.

**Table 1:** **Mothers' reported emotional expressiveness in mother–child interactions (positive and negative), children's sensitivity to criticism (emotional response, rating of ability, and motivation), vocabulary scores, and age: descriptive statistics and correlations**.

		**MOTHER**		**CHILD**				
		**Positive**	**Negative**	**SC_ER**	**SC_AR**	**SC_M**	**VOC**	**Age**
**MOTHER**								
**Positive emotion (Positive)**		–	−.34^*^	.16	.14	−.12	−.17	−.18
**Negative emotion (Negative)**		–	–	−.10	.24^†^	−.36^**^	.15	.28^*^
**CHILD**								
**Sensitivity to criticism**								
** Emotional response (SC_ER)**		–	–	–	.23^†^	.34^*^	.01	.08
** Ability rating (SC_AR)**		–	–	–	–	−.05	−.16	−.11
** Motivation (SC_M)**		–	–	–	–	–	−.23^†^	.15
**Vocabulary (VOC)**		–	–	–	–	–	–	.56^**^
**Age**		–	–	–	–	–	–	–
Mean		34.64	20.19	1.58	.39	.81	32.26	5.91
*SD*		3.81	4.04	.80	.49	.39	9.07	.52

† *p* < .*10*.

* *p* < .*05*.

** *p* < .*01*.

### Maternal emotional expressivity as a predictor of children's sensitivity to criticism

Analyses of the correlation between mother-reported emotional expressiveness and children's sensitivity to failure and criticism revealed that more negative maternal expressiveness was marginally significantly correlated with higher self-rated ability, *r* = 0.24, *p* = 0.082, and significantly correlated with lower motivation to persevere, *r* = −0.36, *p* = 0.009, after teacher criticism. No significant correlations were found between mothers' negative emotional expressiveness and children's sensitivity to failure in the absence of criticism. It should be noted that no significant correlations between mothers' positive expressiveness and children's response to failure or criticism were observed.

To explore the relationship between maternal reports of emotional expressiveness in mother–child interactions and children's sensitivity to criticism after controlling for children's receptive vocabulary and age (in months), two hierarchical multiple regression analyses were conducted with the child's self-rated ability and motivation to persevere following criticism as dependent variables. As the independent variables in each equation, the child's receptive vocabulary score (PVT-R) and age were entered in Step 1, and scores for maternal positive and negative emotional expressiveness were entered in Step 2.

#### Children's ability rating after criticism

As shown in Table 2, children's receptive vocabulary and age, entered in Step 1, were not significantly related to self-rated ability after criticism *F*_(2, 50)_ = 0.70, *p* = 0.503. In Step 2, mother's emotional expressiveness accounted for 15% of the variance, Δ*F*_(2, 48)_ = 3.50, *p* = 0.038, and only negative emotional expressiveness made a unique positive contribution (β = 0.37, *p* = 0.015), suggesting that children with mothers who expressed more negativity rated their abilities more highly following teacher criticism.

**Table 2:** **Mothers' reported emotional expressiveness in mother–child interactions as a predictor of children's sensitivity to teacher criticism**.

	**Children's sensitivity to teacher criticism**					
	**Ability rating**			**Motivation**		
**Predictors**	β	**Total *R*^2^**	**Δ*R*^2^**	β	**Total *R*^2^**	**Δ*R*^2^**
**Step1**		.03			.05	
Child vocabulary	−.15			.21		
Child age	−.03			.03		
**Step2**		.15	.12^*^		.23	.17^**^
Child vocabulary	−.12			.21		
Child age	−.11			−.16		
Maternal positive emotion	.22			.04		
Maternal negative emotion	.37^*^			−.42^**^		

* *p* < .*05*.

** *p* < .*01*.

#### Children's motivation after criticism

As shown in Table 2, children's receptive age and vocabulary, entered in Step 1, were also not significantly related to perseverance after criticism *F*_(2, 50)_ = 1.43, *p* = 0.240. In Step 2, maternal emotional expressiveness accounted for 23% of the variance, Δ*F*_(2, 48)_ = 5.33, *p* = 0.008, and only mother's negative emotional expressiveness made a unique negative contribution (β = −0.42, *p* = 0.004), suggesting that children with mothers who expressed more negativity exhibited lower motivation after teacher criticism.

## Discussion

This study investigated the association between maternal emotional expressiveness in the context of mother–child interactions and young children's sensitivity to teacher criticism. To assess young children's sensitivity to criticism, their patterns of emotional responses, ratings of their own abilities, and motivation following criticism were measured using story-based tasks, similar to those developed by Cutting and Dunn (2002) and Heyman et al. (1992), in which a puppet representing the child made a small mistake and a puppet teacher pointed out that mistake.

The first hypothesis, which predicted that children of mothers who tended to express positive emotions would be less sensitive to teacher criticism, was not supported. No relationships were observed between the extent to which mothers reported expressing positive emotions to their children and their children's sensitivity to criticism. Consistent with studies conducted by Dweck (1999; Kamins and Dweck, 1999), the results of the current study suggest that too much positive feedback or the wrong kind of praise (i.e., person praise) may not have a positive effect on children's motivation. It may be that children who are exposed to a steady stream of too much positive feedback in interactions with their mothers come to expect only positive responses and may not have a chance to develop strategies for coping with negative feedback.

The second hypothesis, which predicted that the children of mothers who tended to express negative emotions would be more sensitive to teacher criticism, was partly supported. The correlation (see Table 1) and multiple regression (see Table 2) analyses suggested that children with mothers who reported expressing more negativity rated their abilities more highly and exhibited less motivation following teacher criticism. No relationships were found between maternal emotional expressiveness and children's sensitivity to failure in the absence of criticism, as manifested in the children's emotional responses, self-rated ability, and motivation to persevere.

These results lead to two important suggestions. First, high self-rated ability does not necessary imply that a child is emotionally stable or has high self-esteem, especially when the rating is made following criticism (cf. Masten and Coatsworth, 1998). As noted earlier, the results of the current study indicate that children with mothers who expressed more negative emotions tended to rate their own ability more positively following criticism. Hughes et al. (1997) examined the association between idealized or inflated self-perceptions and level of aggression in second- and third-grade children and showed that aggressive children viewed their own personal competence and the quality of their relationships in an unrealistically favorable light. That study implied that repeated threats to the ego (Bushman and Baumeister, 1998; Thomaes et al., 2008) in a familial setting can impair a child's perception of his/her own abilities. Given the findings of previous research, it would not be surprising if children with greater exposure to their mothers' negative emotions used a positive self-evaluation as a defense mechanism. Although the cross-sectional data from the current study support the existence of the unique reaction to criticism, additional longitudinal research is needed to test this hypothesis more thoroughly.

Second, children with mothers who expressed more negative emotion may have wanted to avoid additional opportunities for teacher evaluation by not re-engaging in the same project that attracted the original criticism because they may have believed that this would lead to further negative responses, which could create a crisis for them (e.g., Eisenberg et al., 2001). The results also imply that mothers' emotional expressiveness may influence their children's internalized beliefs concerning the acceptance they deserve or can expect from other adults (i.e., teachers). This study highlighted the effect of maternal emotions on the internal processes of young children by addressing individual differences in sensitivity to criticism. It is interesting that, despite the complex and multifaceted environmental influences on children's social development (Rubin, 1998; Sameroff et al., 1998), the effects of maternal emotional expressiveness were significant. This is especially noteworthy given the fact that the children who participated in this study spent about 8–10 hours per day at their nursery school.

The ways in which caregivers respond to their children in daily life have important implications for children's social and emotional development (Gottman et al., 1997; Eisenberg et al., 1998; McElwain et al., 2007). This study makes a significant contribution to clarifying the influence of the mother–child emotional climate at home on teacher–child communication outside the home. However, this study has three major limitations that should be considered: the use of a single self-report measure to assess maternal emotional expressiveness, the cross-sectional design, and the sample size.

Long-term prospective research with a larger sample and both self-report and observational measures are needed to examine causal relationships involving family environments and children's sensitivity to criticism. Future research should rely on observations of the maternal emotions actually displayed during mother–child interactions in addition to self-report data on emotional expressiveness. Such a direct observational approach should be used to validate the findings of this study. Longitudinal research in this area has just started, and the most recent study has found that parents' use of process praise with their toddlers in the home environment predicted children's endorsement of an incremental framework 5 years later (Gunderson et al., 2013). Moreover, because maternal reports of positive expressivity were relatively high, it is possible that the expression of positive emotions by mothers would have been associated with children's sensitivity to criticism if fewer mothers expressed positive emotion to their children. Additionally, other familial variables may play a role in how children respond to criticism. For example, previous studies have shown that fathers may play a unique role in their children's socio–emotional development (Carson and Parke, 1996; Eisenberg et al., 1996; Denham and Kochanoff, 2002). The quality of the marital relationship may also influence children's emotional development (Barry and Kochanska, 2010). Future research should explore these complex interactions in detail. Research of this sort can reveal more about how early parent–child interactions influence individual differences in motivation when children enter the broader social world.

### Conflict of interest statement

The authors declare that the research was conducted in the absence of any commercial or financial relationships that could be construed as a potential conflict of interest.

## Footnotes

^1^The original version of SEFQ measures expressiveness in the *family* context rather than in the mother–child context.

## Appendix

### The modified version of the self-expressiveness in the family questionnaire

1. Showing contempt for your child's actions. (−)
2. Expressing dissatisfaction with your child's behavior. (−)
3. Praising your child for good work. (+)
4. Expressing anger at your child's carelessness. (−)
5. Sulking over unfair treatment by your child. (−)
6. Blaming your child for family troubles. (−)
7. Putting down your child's interests. (−)
8. Showing dislike for your child. (−)
9. Expressing excitement about your child's future plans. (+)
10. Demonstrating admiration about your child to him/her. (+)
11. Expressing deep affection or love for your child. (+)
12. Spontaneously hugging your child. (+)
13. Expressing momentary anger to your child over a trivial irritation. (−)
14. Snuggling up to your child. (+)
15. Trying to cheer up your child when she or he is sad. (+)
16. Telling your child how happy you are. (+)
17. Threatening your child. (−)
18. Expressing gratitude for a favor from your child. (+)
19. Surprising your child with a little gift or favor. (+)

## Notes

*Note*. (+) indicates the item is on the positive dimension; (−) indicates the item is on the negative dimension.
